# Presence of Probst Bundles Indicate White Matter Remodeling in a Dog With Corpus Callosum Hypoplasia and Dysplasia

**DOI:** 10.3389/fvets.2018.00260

**Published:** 2018-10-22

**Authors:** Adriano Wang-Leandro, Matthias Dennler, Katrin M. Beckmann

**Affiliations:** ^1^Clinic for Diagnostic Imaging, Department of Diagnostics and Clinical Services, Vetsuisse-Faculty Zurich, Zurich, Switzerland; ^2^Neurology Department, Clinic of Small Animal Surgery, Vetsuisse-Faculty Zurich, Zurich, Switzerland

**Keywords:** DTI, canine, axonal bundles, hypernatremia, adipsia, seizures, epilepsy

## Abstract

Corpus callosum abnormalities (CCA) rarely occur in dogs and are related to hypo/adypsic hypernatremia and seizures. Hypoplasia and dysplasia of the corpus callosum (CC) with concomitant lobar holoprosencephaly is the most common variant. It is currently uncertain using conventional MRI if canine CCA reflects the failure of commissural fibers to develop or the failure of the commissural fibers to cross hemispheres. Diffusion tensor imaging was performed in a 4-year-old Staffordshire mix breed dog with CCA and an age-matched healthy Beagle. In comparison to the control dog, CC tractography of the affected dog depicted only axonal tracts corresponding to the temporal CC fibers. The cingulum bundles appeared supernumerary with unorganized architecture, extending into the ipsilateral cerebral cortex, and therefore strongly suggested homology to Probst bundles reported in humans with CCA. The presence of Probst bundles in canine CCA could represent compensatory neuroplasticity-mediated networking and may contribute the fair prognosis reported in affected dogs.

## Background

Corpus callosum abnormalities (CCA) occur sporadically in dogs and are classified into hypoplasia with or without concomitant dysplasia and agenesis (1). Additionally, dogs affected with CCA commonly present a mild degree of lobar holoprosencephaly (HPE) (2). In veterinary medicine, it is currently uncertain if CCA in dogs reflects a failure of commissural white matter tracts to develop or a failure to cross the midline and therefore to connect both cerebral hemispheres (1).

In humans affected by CCA, white matter bundles of the cingulum undergo morphological remodeling, thereby changing their trajectory and allowing them to have a wider connectivity within the ipsilateral cerebral hemisphere (3). These fibers, which extend from the cingulum to different regions of the cerebral cortex are also known as Probst bundles (4).

As the prognosis for dogs affected by CCA is reported to be fair to good, most cases are managed symptomatically and therefore histopathological description of white matter integrity is limited. Consequently, diffusion tensor imaging (DTI), a state-of-the-art MRI sequence that enables *in vivo* quantitative and visual characterization of axonal bundle microarchitecture, may contribute to overcome these limitations (5). Fractional anisotropy (FA; unitless) and mean diffusivity (MD; 10–3 mm^2^/s) values describe the directionality and magnitude of water molecule diffusion within the white matter tracts, respectively (6).

The present case report describes the morphology and integrity of the corpus callosum and cingulum in a dog affected by CCA.

## Case presentation

A 4-year-old, male Staffordshire Terrier mix was presented to the Neurology Department of the Vetsuisse Faculty Zurich due to generalized seizures. The first seizure episodes were reported as a puppy. At this time, severe hypernatremia secondary to adipsia was diagnosed and the dog was successfully managed by adding water to his meals. After the hypernatremia was corrected, no seizure episodes were observed for 3 years thereafter.

Five months before presentation, seizures began to reoccur every second week despite normal serum sodium levels. Moreover, 4 days before presentation phenobarbital (2.5 mg/kg; every 12 h) was started by the referring veterinarian. The general physical examination at presentation was within normal limits and blood work showed only mild hypernatremia (sodium 156 mmol/l; reference: 145–152 mmol/l) and mildly increased serum albumin levels (albumin 42 g/l; reference: 29–37 g/l). The neurological examination revealed proprioceptive deficits in all limbs, proprioceptive ataxia and reduced menace response bilaterally. No visual impairment could be detected during the neurological examination. Therefore, the neuroanatomical localization was consistent with a forebrain dysfunction. CCA was suspected due to the history and clinical presentation.

Afterwards, an MRI of the brain was performed under general anesthesia using a 3T scanner (Philips Ingenia, Philips AG, Zurich, Switzerland). T2-weighted (T2W) sequences were acquired in sagittal (TR 6836 ms; TE 100 ms), transverse (TR 4820 ms; TE 100 ms), and dorsal (TR 3118 ms; TE 100 ms) planes. A 3D T1-weighted (T1W; TR 11.1 ms; TE 5.1 ms) sequence was obtained pre and post intravenous administration of gadodiamid (0.1 mmol/kg; Omniscan, GE Healthcare AG, Opfikon, Switzerland). Supplementary to the conventional protocol, an echo-planar DTI sequence (TR 8534.3 ms; TE 84.3 ms) with 32 diffusion directions (low b value = 0 s/mm^2^; maximal b value = 800 s/mm^2^; field of view of 160 × 169 mm and acquisition matrix of 108 × 111) was performed. Moreover, T2W, 3D-T1W, and DTI sequences from a 4-year-old, intact male healthy Beagle (same acquisition protocols, archived from an independent research study; animal permission number: ZH272/16) were used for comparison.

For the DTI analysis, corrections for drifting, motion artifacts, eddy-currents and Gibbs-ringing were performed. Subsequently, the DTI sequences were registered to the anatomical T1W images. Whole brain tractographies were calculated using the following settings using a probabilistic approach: minimal FA threshold 0.1; minimal fiber length 20 mm and a threshold angle of 60 degrees. Afterwards, regions of interest (ROIs) were placed at the CC within sagittal planes and at the cingulum within transverse planes in order to isolate the axonal tracts of these regions where they solely cross the laterolateral or rostrocaudal axis, respectively, as previously reported (7). The evaluation of T1W, T2W, and the tractography analysis of DTI images were performed using free available software (Horos Project, version 2.2.0, horosproject.org; ExploreDTI, version 4.8.6, Utrecht) (8).

## MRI findings

**Figure 1:**
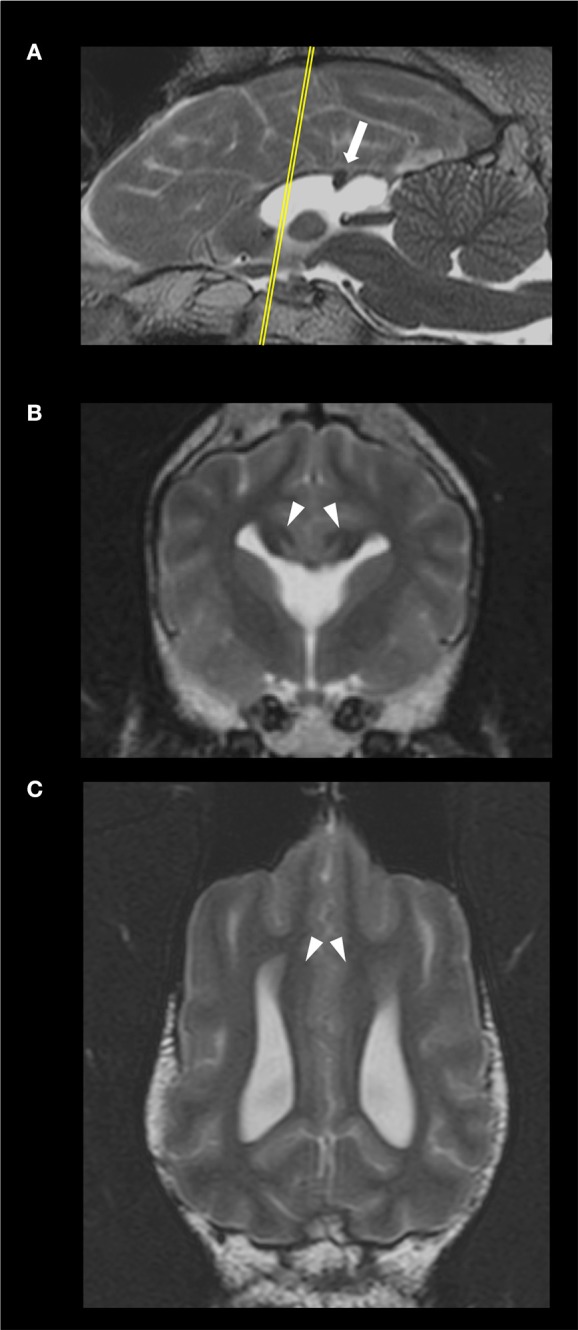
Sagittal **(A)**, transverse, at the level of the optic chiasma **(B)**, and dorsal, at the level of the cingulum **(C)**, T2-weighted images of the 4 year-old, male Staffordshire Terrier mix with hypodipsia and seizures. The yellow line in **(A)** indicates the level at which the transverse plane is depicted. The corpus callosum (CC) is hypoplastic and dysplastic. The white arrow points to a discrete focal area of preserved thickness and white matter signal intensity within the splenium of the CC **(A)**. The arrowheads point at the mildly enlarged cingula, bilaterally **(C)**. The lateral ventricles and rostroventral segments of the frontal lobes are fused; there were no detectable fornix nor septum pellucidum **(B)**.

The MRI of the brain revealed a discrete area of preserved thickness and signal intensity of white matter at the level of the splenium and a non-detectable rostrum, genum and corpus (Figure 1). Additional MRI findings included fusion of the rostroventral region of the frontal lobe, a non-detectable septum pellucidum with associated fusion of the lateral ventricles, absence of the fornix and dilation of the pineal recess. The cingula of the affected dog appeared mildly increased in thickness bilaterally. Therefore, according to a previously established classification of CCA in dogs (1), the diagnosis of hypoplasia and dysplasia of the CC with concurrent mild lobar HPE and mild enlargement of the cingula was made.

**Figure 2:**
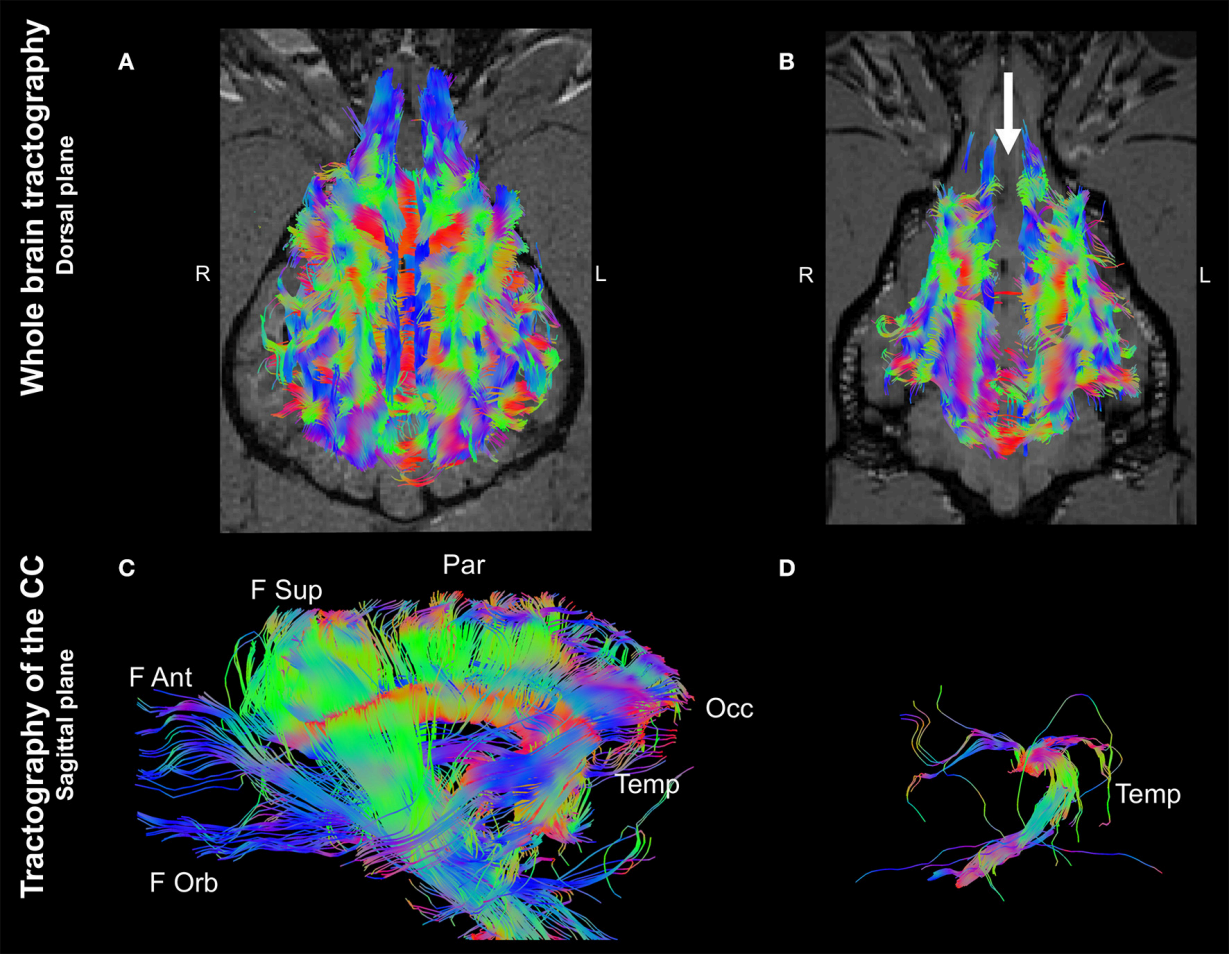
Whole brain tractographies registered to T1-W images at the level of the caudal colliculi depicted in a dorsal plane and corpus callosum tractographies in the sagittal plane (rostral to the left) of a 4-year-old, male healthy Beagle **(A,C)** and the 4-year-old, male Staffordshire Terrier mix with corpus callosum abnormalities (CCA; **B,D**). The majority of axonal bundles crossing hemispheres are absent in the dog with CCA (white arrow; **(B)** in comparison to the healthy Beagle. Tractography of the CC revealed that only temporal fibers were present, corresponding to the discrete area of remaining white matter noticed in T2-weighted sequences **(D)** Color coding of tracts: Red, laterolateral axis; green ventrodorsal axis; blue, rostrocaudal axis. F Ant, frontal anterior; F Orb, frontal orbital; F Sup, frontal superior; Occ, occipital; Par, parietal; Temp, temporal.

Whole brain tractographies revealed a generalized absence of CC commissural fibers in the dog with CCA in comparison to the healthy control. The tractography of the healthy control displayed morphological consistency with previous reported literature describing the normal CC (9, 10) (Figures 2A,**B**). Furthermore, tractographies originating from ROIs placed at the CC in the control dog revealed the presence of frontal orbital, anterior and superior parietal, occipital, and temporal CC fibers; whereas only temporal fibers could be depicted after analyzing the same ROIs in the dog affected by CCA (Figures 2C,D).

**Figure 3:**
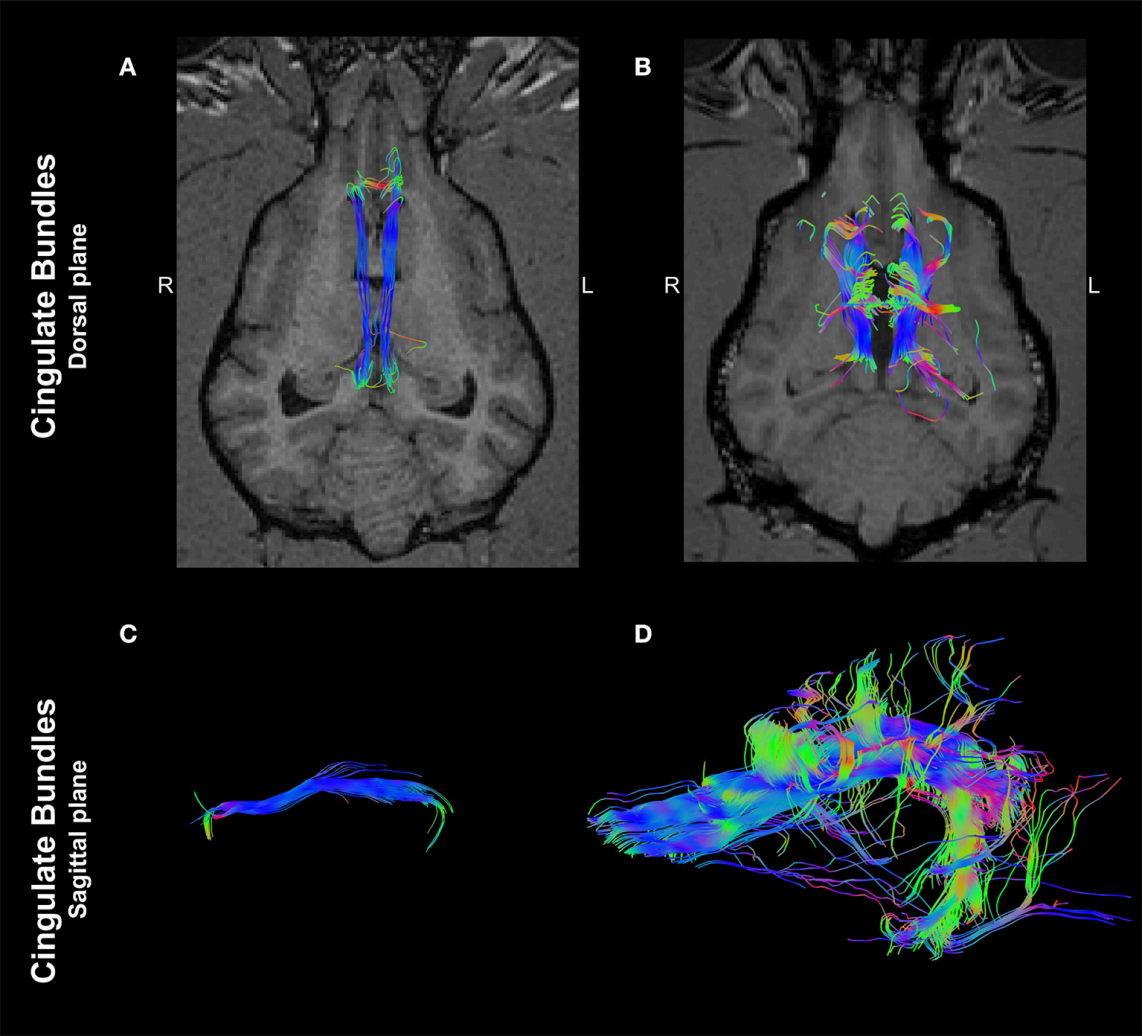
Tractographies derived from regions of interest positioned at the cingulum of a 4-year-old, male healthy Beagle **(A,C)** and the 4-year-old, male Staffordshire Terrier mix with corpus callosum abnormalities (CCA; **B,D**). The tractographies are shown in the dorsal plane, registered to T1-W images at the level of the thalamus **(A,B)** and in the sagittal plane (rostral to the left; **C,D**). Supernumerary axonal bundles with unorganized morphology projecting to the ipsilateral cortex are present in the dog affected by CCA in comparison to the control dog. Color coding of tracts: Red, laterolateral axis; green, ventrodorsal axis; blue, rostrocaudal axis.

Interestingly, an analysis of ROIs placed at the cingulum depicted a marked morphological difference between the two dogs. In the control dog, tractography of the cingulum appeared as discrete, parallel and symmetrical fibers crossing along the rostrocaudal axis (Figures 3A,C). In contrast, tractography derived from the same localization of the dog affected with CCA revealed supernumerary fibers displayed in an unorganized morphology, projecting in the ventrodorsal axis to the ipsilateral cerebral cortex (Figures 3B,D).

Values of FA and MD from the tractography analysis of each ROI are shown in Table 1.

**Table 1:** DTI metrics of tracts derived from regions of interest placed at corpus callosum and cingulate bundles.

	**DTI metrics**	**Dog with CCA**	**Control**
Corpus Callosum	FA	0.287 (splenium)	0.326
	MD (10^−3^ mm^2^/s)	1.29 (splenium)	1.03
Cingulum	FA	0.321	0.305
	MD (10^−3^ mm^2^/s)	0.989	0.857

*DTI, diffusion tensor imaging; CCA, corpus callosum abnormalities; FA, fractional anisotropy; MD, median diffusivity*.

Following the MRI examination medical treatment with phenobarbital was continued and the dosage was adjusted based on the phenobarbital blood levels. Six months after the MRI scan, the owners were contacted by telephone for a follow up. Seizure frequency decreased during the first 5 months after the initiation of treatment; however, two episodes of cluster seizures occurred despite adequate phenobarbital blood levels during the fifth month. Levetiracetam was started as an add-on treatment at a dosis of 20 mg/kg every 8 h. At the time of writing this manuscript (3 months later) the dog has remained seizure free.

## Discussion

CCA occurs rarely in dogs and the most common associated clinical signs include adipsia/hypodipsia and seizures (1, 11–13). Staffordshire Terriers and Miniature Schnauzers are the most commonly reported affected breeds (1, 11). Adipsia/hypodipsia has been attributed to concomitant HPE present in dogs affected with CCA, due to the fact that osmoreceptors for plasma and CSF are present in rostral commissural structures (12, 13). Consequently, hypernatremia is a common finding in affected dogs and culminates in reactive seizures caused by electrolyte imbalances (13). However, in the present case, the dog with CCA showed seizures with subclinical levels of hypernatremia. As the presence of CCA does not *per se* imply the occurrence of seizures, an alternative mechanism has to be considered. An epileptic focus secondary to the frontal cortex anomaly present in HPE, as described in humans with CCA, represents the most likely independent or parallel cause of seizure development (14).

In the present case, the CC is considered to be hypoplastic and dysplastic, with a partial agenesis of the rostral portion and a simultaneous discrete focal area of apparent preserved thickness of the splenium (1, 9). Furthermore, the DTI visualizes the presence of crossing temporal CC fibers that correspond to the focal area of preserved white matter signal intensity noticed in conventional T2W sequences. In contrast, the CC tractography of the control dog displayed the six major white matter tracts (frontal orbital, frontal anterior, frontal superior, parietal, occipital and temporal), consistent with previously described CC segmentation by means of DTI (9). A minimal FA threshold of 0.1 was intentionally set to allow detection of possible degenerated white matter tracts of the CC crossing in the laterolateral axis. Interestingly, FA and MD values of the fibers crossing between hemispheres showed similar behavior in both dogs. This is in agreement with previously reported normal values for CC acquired with a 3T magnet and a similar acquisition protocol (7). This finding indicates preserved microarchitecture of the white matter and suggests that DTI represents a useful method to differentiate CCA from degenerative diseases causing morphologic changes of the CC (15).

A marked morphological difference of the cingulum bundles between the dog affected with CCA and the control dog is present. In the affected dog, these fibers are supernumerary and show a disorganized conformation, extending to the ipsilateral cerebral cortex. In humans affected by CCA, remodeled cingulum bundles have been comprehensively described using DTI tractography (4, 5, 16). Among humans affected by CCA, compensatory neuroplasticity of the ipsilateral hemispheric axonal tracts is proposed as the mechanism responsible for remodeling of the cingulum and a variable degree of Probst bundle development is evident among patients (17). This aspect remains uncertain for canine CCA, as the present report is based on a single case; however, this presents an opportunity to prospectively screen for the presence and characteristics of Probst bundles using DTI.

Although the exact function of the Probst bundles remains unknown, a recent study combining diffusion tensor MRI and electroencephalography (EEG) correlated the presence of Probst bundles with intrahemispheric long distance EEG coherence, therefore suggesting an aberrant functional connectivity (18).

To the authors' knowledge, this is the first report of the presence of Probst bundles in a dog with CCA. This case report highlights the importance of the clinical application of DTI in enhancing the understanding of white matter remodeling secondary to CCA that cannot be detected with conventional MRI sequences.

## Ethics statement

The manuscript represents a report of a single clinical case presented to a referral teaching veterinary hospital; therefore, no ethical approval was needed. The owner signed a consent form that the dog's data can be used for scientific studies.

## Author's note

Data contained in this case report was partially presented at the 14th European MRI User Meeting, Zurich, April 28–29th 2018 and at the 31st Annual Symposium of the ESVN-ECVN, Copenhagen, Denmark, September 20th–22nd 2018 as an oral and poster presentation, respectively.

## Author contributions

AW-L performed the MRI interpretation and analysis and wrote the manuscript. MD was involved in the methodology of image acquisition and interpreted the MR images. KB supervised the clinical management of the case. All authors critically reviewed and approved the final version of the manuscript.

### Conflict of interest statement

The authors declare that the research was conducted in the absence of any commercial or financial relationships that could be construed as a potential conflict of interest.

## Abbreviations

CC: Corpus callosum
CCA: Corpus callosum abnormalities
CSF, Cerebrospinal fluid, DTI: Diffusion tensor imaging
EEG, Electroencephalography, FA: Fractional anisotropy
HPE: Holoprosencephaly
MD: Mean diffusivity
MRI: Magnetic resonance imaging
ROI: Region of interest
T: Tesla
T1W: T1-weighted
T2W: T2-weighted
TE: Echo time
TR: Repetition time.
